# An Experimental Novel Study: *Angelica sinensis* Prevents Epidural Fibrosis in Laminectomy Rats via Downregulation of Hydroxyproline, IL-6, and TGF-**β**1

**DOI:** 10.1155/2013/291814

**Published:** 2013-08-29

**Authors:** Chao Zhang, Xiaohong Kong, Hengxing Zhou, Chang Liu, Xuechao Zhao, Xianhu Zhou, Yanhua Su, Hari S. Sharma, Shiqing Feng

**Affiliations:** ^1^Department of Orthopedics, Tianjin Medical University General Hospital, Tianjin 300052, China; ^2^School of Medicine, Nankai University, Tianjin 300071, China; ^3^Tianjin Medical Universtiy, Tianjin 300070, China; ^4^Laboratory of Cerebrovascular Research, Department of Surgical Sciences, Anesthesiology and Intensive Care Medicine, University Hospital, Uppsala University, Frödingsgatan 12, 75421 Uppsala, Sweden

## Abstract

With laminectomy being widely accepted as the treatment for lumbar disorders, epidural fibrosis (EF) is a common complication for both the patients and the surgeons alike. Currently, EF is thought to cause recurrent postoperative pain after laminectomy or after discectomy. *Angelica sinensis* is a traditional Chinese medicine which has shown anti-inflammatory, antifibrotic, and antiproliferative properties. The object of this study was to investigate the effects of *Angelica sinensis* on the prevention of post-laminectomy EF formation in a rat model. A controlled double-blinded study was conducted in sixty healthy adult Wistar rats that underwent laminectomy at the L1-L2 levels. They were divided randomly into 3 groups according to the treatment method, with 20 in each group: (1) *Angelica sinensis* treatment group, (2) saline treatment group, and (3) sham group (laminectomy without treatment). All rats were euthanized humanely 4 weeks after laminectomy. The hydroxyproline content, Rydell score, vimentin cells density, fibroblasts density, inflammatory cells density, and inflammatory factors expressions all suggested better results in *Angelica sinensis* group than the other two groups. Topical application of *Angelica sinensis* could inhibit fibroblasts proliferation and TGF-**β**1 and IL-6 expressions and prevent epidural scar adhesion in postlaminectomy rat model.

## 1. Introduction

Laminectomy is widely accepted across the medical community as the treatment of choice in lumbosacral disorders. As early as 1995, researchers reported as many as over one million patients worldwide who underwent lumbosacral surgery to treat disc herniation [1]. Subsequently, the literature reports that patients could suffer from continued pain in the lower posterior trunk and/or lower extremities after lumbar laminectomy, this is commonly referred to as the “failed back surgery syndrome” (FBSS) [2]. Some researchers pointed out that EF is a contributing factor for FBSS in a significant subset of patients [3].

EF is fibrous tissue deposit in the epidural space following posterior spinal surgery [2, 4], first mentioned in 1948 [5]. Until the present, there are no effective treatments with an established EF, and the success rates of reoperations for FBSS are poor [6]. It seems that minimally invasive surgical technique (MIST) is the most commonly used preventative measures against complication. However, among lumbar disorder patients, MIST are not suitable for every patient. Most authors believe that the best way for preventing the occurrence of EF is to prevent its formation [7]. Recently, extensive studies on this have been conducted on animals with several differing components, such as autologous fat grafts, Adcon-L, animal collagen membranes, Gelfoam, anti-inflammatory agents, fibrinolytic agents, honey, and others [8–13]. However, an optimal solution has yet to be discovered or published.

Recently, researchers are putting their focus on the effect of traditional Chinese medical agents with EF suppressing properties, such as *Panax notoginseng (Burkill) F.H. Chen ex C.H. Chow (Araliaceae)* sodium hyaluronate gel which demonstrated prevention of epidural scar adhesion in a rat model setting [14]. We discovered that the *Angelica sinensis (Oliv.) Diels (Apiaceae)* agent demonstrated curative properties as well in laminectomy rat. 

The root of *A. sinensis*, known as *Danggui* in Chinese, is a well-known traditional medicine that is widely used in China for gynecological diseases with clinical efficacy [15]. Extracts and compounds purified from *A. sinensis* roots were in extensive use for increasing myocardial blood flow and reducing radiation induced tissue damages [16–18]. At the same time, with a series of advantages of little or no toxicity, *A. sinensis* has been used in treating cancer patients and has shown clinical efficacy [19]. 

In our rat laminectomy model, we investigated whether *A. sinensis* attenuates EF by regulating the expressions of interleukin 6 (IL6), transforming growth factor-*β*1 (TGF-*β*1), and hydroxyproline, which are suggested to be involved in the promotion and/or development of EF.

## 2. Material and Methods

### 2.1. Animals

Sixty healthy male Wistar rats (mean weight = 250 g) were purchased from Radiation Study Institute-Animal Center, Tianjin, China. The animals received care in compliance with the principles of International Laboratory Animal Care, and this study was performed by the approval of the Animal Care and Research Committee of Tianjin Medical University. All rats were randomly divided into three groups (twenty rats per group): (1) *A. sinensis* treatment group, (2) saline treatment group, and (3) sham group (laminectomy without treatment).

### 2.2. Reagents and Antibodies

*A. sinensis* extract was provided by the First Hospital Affiliated to Tianjin University of Traditional Chinese Medicine. *β*-Dimethylaminobenzaldehyde was purchased from Sigma Corporation. Cal-EX II solution for decalcification and dehydration was purchased from Fisher Scientific, Orangeburg. Reverse Transcriptase was purchased from Promega, Madison, Wisconsin. Primary antivimentin antibody (ab92547) was purchased from abcam. Secondary antibodies were purchased from Santa Cruz. 

### 2.3. Laminectomy Rat

**Figure 1:**
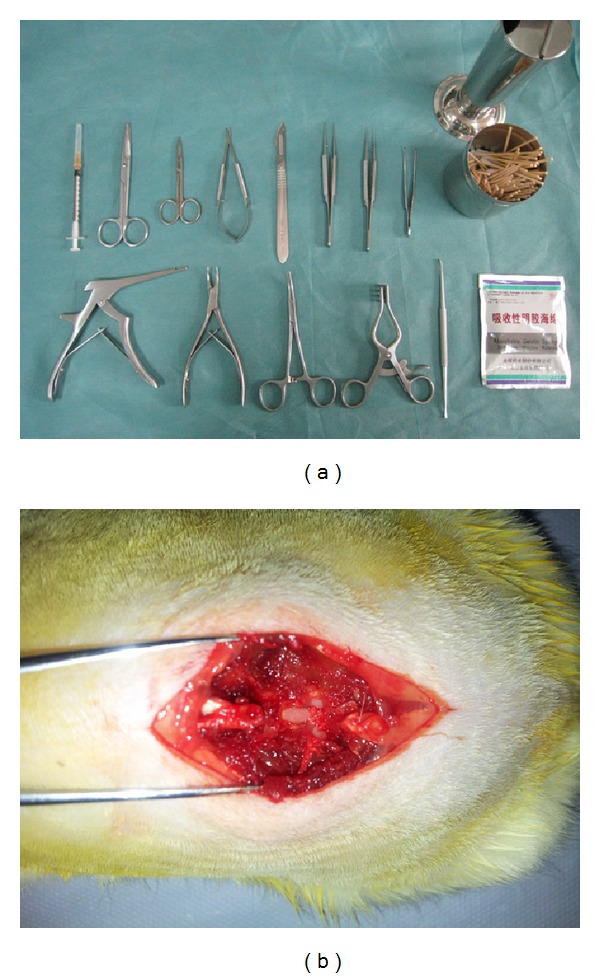
Preoperative preparation, rat laminectomy. (a) Requisite intraoperative surgical instruments. (b) Laminectomy was conducted at L1 level.

Laminectomy was performed under sterile conditions employing basic surgical tools (Figure 1(a)). Each rat was numbered. All rats were anesthetized by intra-peritoneal injection of 10% chloral hydrate (0.3 mL/100 g body weight) and restrained on a warm pad in the prone position. The hairs around the L1 and L2 were shaved, and the exposed skin was sterilized. Our total laminectomy model was based on the previous study [18]. A median incision of dorsal skin was made and the paraspinal muscles were separated on L1-L2 vertebrae. The dura mater of L1 vertebrae was exposed after removing the spinous process and vertebral plate with a rongeur (Figure 1(b)). Gauze was used for homeostasis. Close attention was paid not to traumatize the dura and the nerve roots.

### 2.4. Topical Application of *A. sinensis*

*A. sinensis* or saline was applied to the laminectomy sites with cotton pads (5 × 4 mm^2^, application volume is about 0.8 mL) for 5 min separately. Wet gauzes were used to cover the surrounding tissues to prevent the contact of agent. After the cotton pads were removed from the surgical field, the laminectomy sites were irrigated immediately with saline to eliminate surplus *A. sinensis*. Then, the wound site was surgically closed.

### 2.5. Macroscopic Assessment of EF

Macroscopic assessment was performed four weeks postoperatively. Five rats were randomly selected from each group and anesthetized. The surgical sites were reopened and the epidural scar adhesion underwent double blind evaluation and the results were classified based on the Rydell classification (Table 1) [20]. 

### 2.6. Determination of Hydroxyproline Content (HPC) in Epidural Scar Tissue

Hydroxyproline content (HPC) analysis was performed four weeks postoperatively. Five rats were euthanized humanely with chloral hydrate. The scar tissue approximately 5 mg wet weight was obtained around the laminectomy site. The content of hydroxyproline of different groups in scar tissue was examined according to the protocol of previous study [21]. The samples were lyophilized, ground, and hydrolyzed with 6 mol/L HCl at 110°C for 24 hours. Then, 1 mL hydroxyproline developer (*β*-dimethylaminobenzaldehyde solution) was added to the samples and the standards. The absorbances at 550 nm were read using a spectrophotometer. Finally, the hydroxyproline content (HPC) per milligram of scar tissue was calculated according to the standard curve constructed by the serial concentration of commercial hydroxyproline.

### 2.7. Histological Analysis

Histological analysis was performed four weeks postoperatively. Five rats selected randomly were euthanized humanely. After that intracardial perfusion with 4% paraformaldehyde was performed. The whole L1 vertebral column including the paraspinal muscles and epidural scar tissue was resected en bloc and fixed in 10% phosphate-buffered formaldehyde solution. After decalcification and dehydration with Cal-Ex II solution for 2 days, they were embedded in paraffin, and 5 *μ*m axial sections of the laminectomy site were stained with hematoxylin and eosin (H&E). The epidural scar adhesion was evaluated under the light microscope. Three counting areas were selected at the middle and at the margins of the laminectomy sites. The number of fibroblasts and inflammatory cells was calculated according to previous study [22] as follows: the cells in three different areas were counted and mean was calculated. The fibroblast and inflammatory cell densities were graded. Grade 1, less than 100 fibroblasts/inflammatory cells per ×400 field; Grade 2, 100 to 150 fibroblasts/inflammatory cells per ×400 field; Grade 3, more than 150 fibroblasts/inflammatory cells per ×400 field. To be more stringent, in order to quantify the fibroblasts numbers, the immunohistochemistry was performed with application of the monoclonal antivimentin antibody, and the density of vimentin was evaluated.

### 2.8. Analysis of IL-6 and TGF-*β*1 Concentrations

The mRNA analyses of IL-6 and TGF-*β*1 were performed four weeks postoperatively. Five rats were euthanized humanely, and the scar tissues from the laminectomy sites were collected. Total RNA was extracted using TRIzol reagent, and the RNA (2 *μ*g) was transcribed into cDNA by use of AMV reverse transcriptase. Quantitative real-time PCR (RTPCR) was performed according to previous study using the Bio-Rad MYIQ2 (USA) [23]. Primer sequences are as follows: TGF-*β*1 (148 bp): forward, 5′-GCCCTGCCCCTACATTTGG-3′, reverse, 5′-CTTGCGACCCACGTAGTAGACGAT-3′, IL-6 (131 bp): forward, 5′-ACCCCAACTTCCAATGCTCT-3′, reverse, 5′-TGCCGAGTAGACCTCATAGTGACC-3′; GAPDH (169 bp): forward, 5′-TCACCACCATGGAGAAGGC-3′; reverse, 5′-GCTAAGCAGTTGGTGGTGCA-3′. GAPDH amplification was used as an internal control.

### 2.9. Statistical Analysis

Data are expressed as mean ± SEM values of the mean, median, and minimum-maximum. Statistical analyses of the data were performed by variance analysis using SPSS 13.0 statistical package. Statistical significance was determined at *P* < 0.05.

## 3. Results

### 3.1. Macroscopic Assessment of Epidural Scar Adhesion

The surgery was well tolerated by all animals without any sign of wound infection, neurological deficit, and cerebrospinal leak. The recovery of all rats was uneventful after the operations. Macroscopic observation showed that soft or weak fibrous adhesion was seen in the laminectomy sites in the *A. sinensis* group. However, severe epidural adhesions were observed around the laminectomy sites in the saline group and sham group, which were difficult to dissect the scar adhesions accompanied with bleeding and disruption of the dura mater. The grades of epidural scar adhesion in rats were evaluated according to Rydell's classification (Table 2). 

### 3.2. Hydroxyproline Content (HPC)

**Figure 2:**
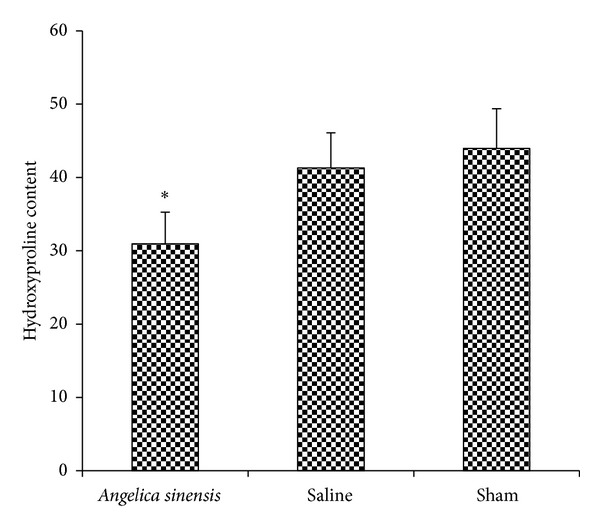
Hydroxyproline levels were expressed as the mean ± standard deviation of hygro tissue. The *A. sinensis* group showed the least hydroxyproline level. *P < 0.05, compared with other two groups.

The HPC of epidural scar tissue in each group was shown in Figure 2. The HPC in the *A. sinensis* group was less than those of the saline group (*P* = 0.003) and sham group (*P* = 0.001). The content in saline group showed no significant difference compared with that of sham group (*P* = 0.095).

### 3.3. Histological Analysis

**Figure 3:**
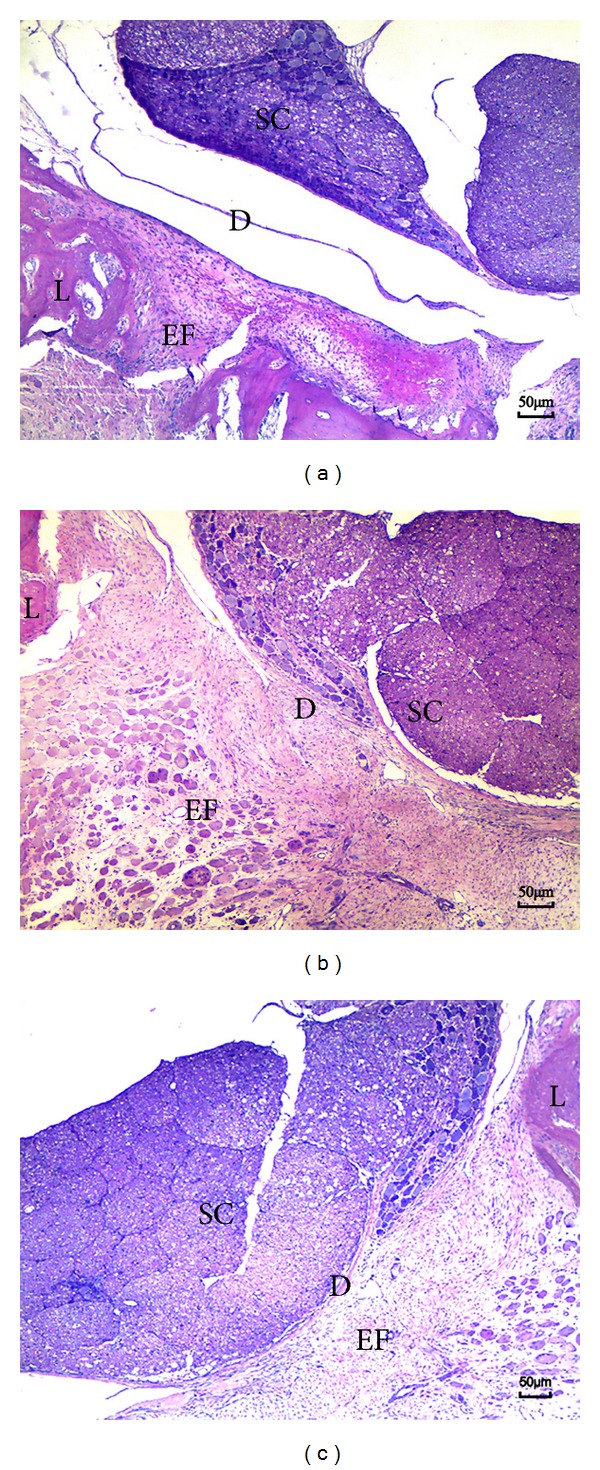
H&E staining for the epidural adhesion tissues in the laminectomy sites applied with *A. sinensis* (a), saline (b), and nothing (c). (a) Loose scar tissues without adherence to dura mater were found in *A. sinensis* group. (b), (c) Dense scar tissues adhered to dura maters were found in saline and sham group. S: spinal cord, L: laminectomy defect, D: dura mater, and EF: epidural fibrosis.

**Figure 4:**
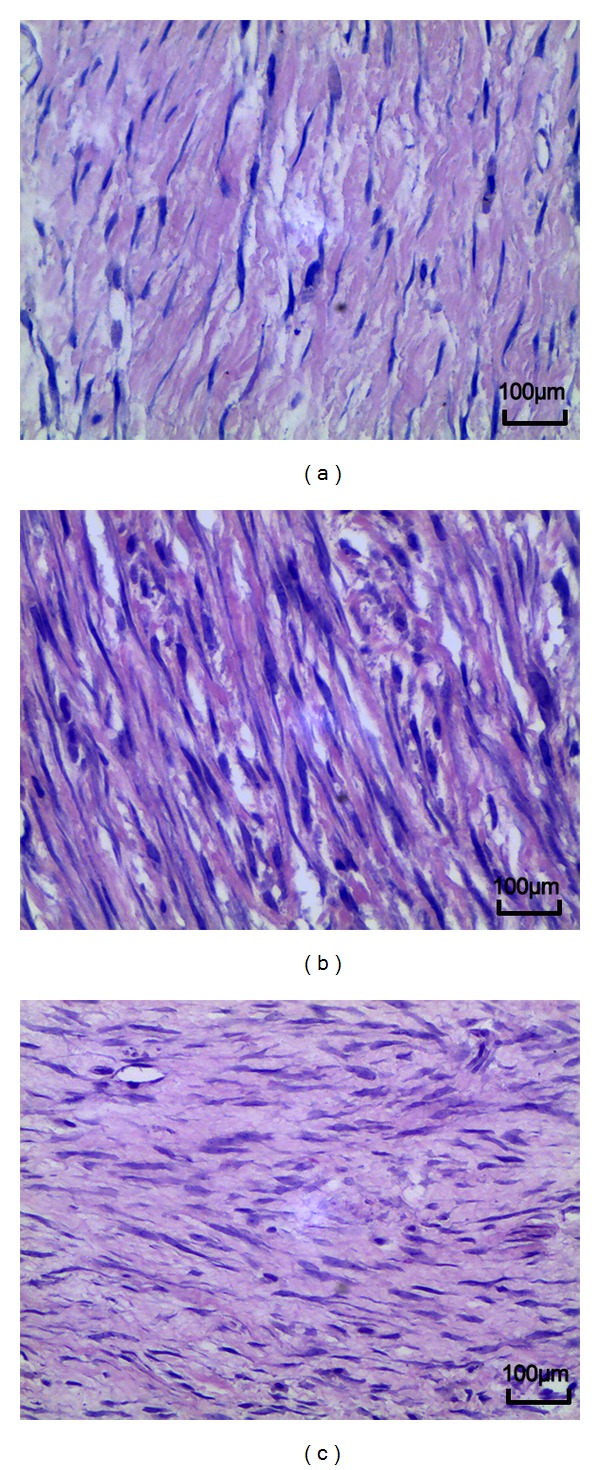
H&E staining analysis of fibroblast in epidural scar tissues applied with *A. sinensis* (a), saline (b), and nothing (c). The density of fibroblast in the *A. sinensis* group (a) was less than those of the other 2 groups. The density of fibroblasts in the saline group was similar with that of the sham group.

In the saline group and sham group, dense epidural scar tissue with widespread adhesions to dura mater and dorsal muscle was observed in the laminectomy sites (Figures 3(b) and 3(c)). A large quantity of fibroblasts appeared in the scar tissue around the laminectomy sites (Figures 4(b) and 4(c)). However, loose or little scar adhesion was observed in the laminectomy sites in *A. sinensis* group (Figure 3(a)), and the number of fibroblasts was significantly less than those of the saline and sham groups (Figure 4(a)). 

### 3.4. Effect of *Angelica sinensis* on Fibroblasts and Inflammatory Cells

**Figure 5:**
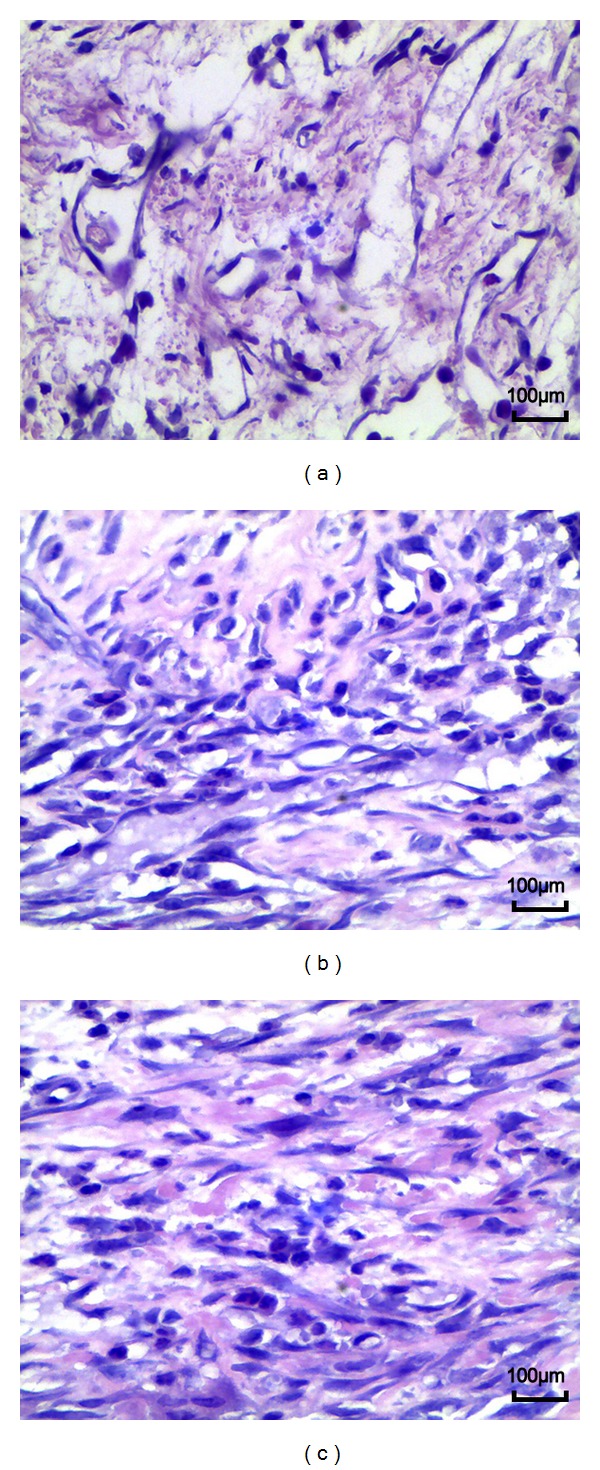
H&E staining analysis of inflammatory cells in epidural scar tissues applied with *A. sinensis* (a), saline (b), and nothing (c). The density of inflammatory cells in the *A. sinensis* group (a) was less than those of the other 2 groups. The density of inflammatory cells in the saline group was similar with that of the sham group.

The fibroblasts and inflammatory densities grades of epidural scar tissue in each group are set out in Table 3. The fibroblasts density in *A. sinensis* group was less than those of the saline group and sham group (Figure 4). At the same time, the inflammatory cells density in *A. sinensis* group was less than those of the saline group and sham group (Figure 5). Both fibroblasts and inflammatory cells densities did not show a significant difference between the saline group and sham group.

**Figure 6:**
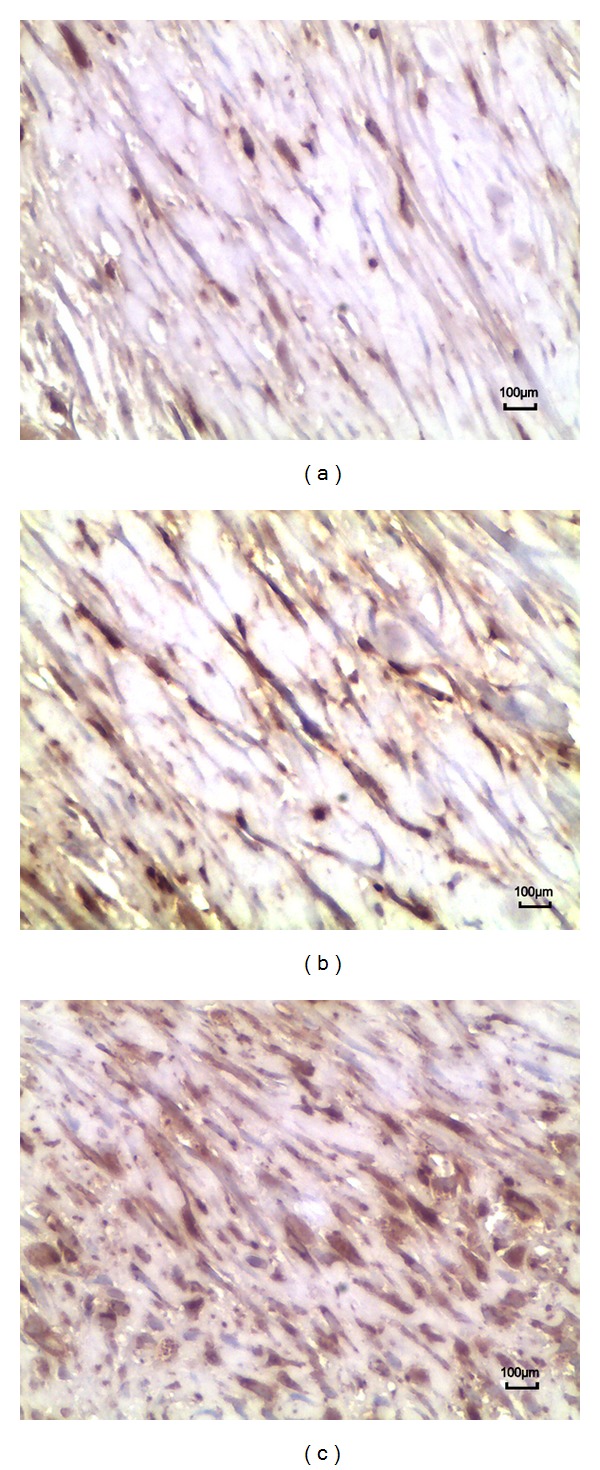
Immunohistochemistry analysis of vimentin cells in epidural scar tissues applied with *A. sinensis* (a), saline (b), and nothing (c). The density of vimentin cells in the *A. sinensis* group (a) was less than those of the other 2 groups. The density of vimentin cells in the saline group was similar with that of the sham group.

In our effort to be more definitive, we conducted an additional immunohistochemistry analysis for vimentin; our data showed that less vimentin was observable in the *A. sinensis* group versus the saline and sham groups (Figure 6).

### 3.5. Effect of *Angelica sinensis *on IL-6 and TGF-*β*1

**Figure 7:**
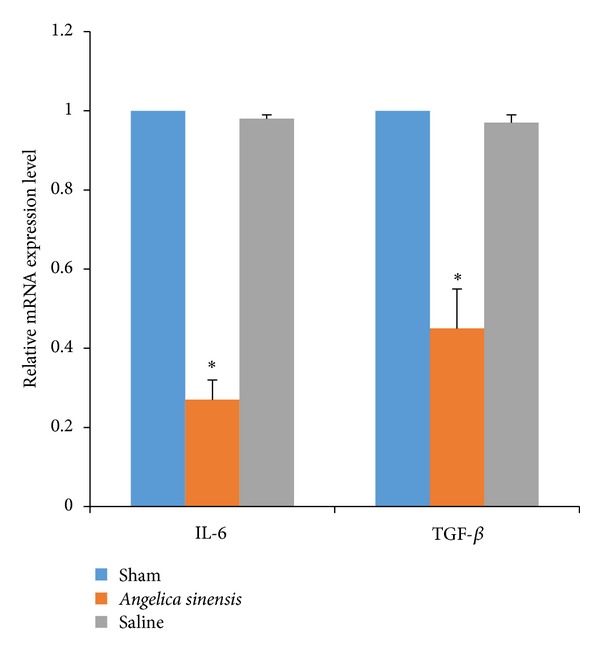
The mRNA expression level of IL-6 and TGF-*β*1 in epidural scar tissue in each group. RT-PCR testing was conducted to evaluate. *P < 0.05, compared with sham group.

In our study employing EF rats, *A. sinensis* has shown the ability to suppress the expressions of TGF-*β*1 and IL-6. Recent studies also mention that *A. sinensis* has the ability to suppress the expression of TGF-*β*1 in lung cancer study [24] and the expressions of TNF-*α* and IL-6 in chronic inflammatory diseases [25]. These studies inspired us to determine whether *A. sinensis* plays a similar role with TGF-*β*1 and IL-6 in EF rats. In order to find out, we conducted RTPCR to examine the mRNA expression levels of both TGF-*β*1 and IL-6. The results of mRNA expression levels of both TGF-*β*1 and IL-6 are shown in Figure 7: the *A. sinensis* group was lower than those of the saline group (*P* = 0.011) and sham group (*P* = 0.009); the expressions between saline group and sham group did not show significant differences (*P* = 0.077). 

## 4. Discussion

FBSS is one of the most common complications encountered in the posterior approach laminectomy, often negatively impacting the surgical outcome. Historically, the literature reports that the incidence of FBSS for lumbar discectomy patients was in the range of 8–40% [26]. The progression of EF has been linked to likely increase one's chance of developing lower posterior trunk or lower extremities pain in as many as 25% of all FBSS [27]. The formation of EF is a complex process and is mainly mediated by the body's inflammatory response. EF develops as a result of invasion of the postoperative hematoma by the dense fibrotic tissue which develops on the level of the fibrous periosteum and the deep paravertebral muscles. This fibrosis can extend to the nodal canal and can adjoin the nerve root and dura mater [28]. Patients will usually not complain of FBSS until about 3–6 months postoperatively; also the onset of pain is usually insidious [26]. EF is characterized by the accumulation of fibroblasts, the depositing of extracellular matrix proteins, and the distortion of normal tissue architecture with inflammation [29]. Therefore, it is important to reduce the three characterizing factors in order to effectively curtail EF.

*A. sinensis* has demonstrated high efficacy in multifaceted suppression of the aforementioned factors. The data showed significant decrease of hydroxyproline levels in scar tissues. In the laminectomy rat, both the inflammatory cells and fibroblast cells decreased significantly after topical application of *A. sinensis*. The scar adhesions showed better Rydell classification scores when treated with *A. sinensis*. RTPCR analyses showed a significant decrease of both IL-6 and TGF-*β*1. All of the above support our theory of *A. sinensis*'s antifibrotic, anti-inflammatory, and antiproliferative roles. In addition, and literatures within the medical community also support our theory by reporting on *A. sinensis*'s anti-fibrotic, anti-inflammatory, and antiproliferative properties [24, 25, 30]. Pharmacological studies have shown that *A. sinensis* extracts are able to improve local and systemic blood flow, which provides benefits in patients with ulcer, cancer, and other diseases. These studies and our data may explain some if not all of the possible mechanisms that make *A. sinensis* effective in preventing or suppressing EF. 

The formative process of EF after laminectomy is a complicated process which may involve multisignaling pathways. Therefore, it could be difficult for single material or agent application to effectively prevent EF. We hypothesize that along with the development of various newly discovered materials and agents, especially some traditional Chinese native medicines, EF could be significantly suppressed with the application of these newly discovered materials along with conjunctive use of *A. sinensis*.

After extensive literature reviews, we feel confident to report that our findings may be one of the first studies looking into *A. sinensis*'s suppressive effects on EF by downregulating the expression of TGF-*β*1 and IL-6 and also reducing hydroxyproline deposition in rats. In our rat EF study, we did not attempt to define neither the most effective dose nor high dose toxicity. Undoubtedly, more research on drug safety, effectively safe concentration, long-term effects, and possible side and adverse effects of *A. sinensis* are all warranted before clinical trials and application.

## Figures and Tables

**Table 1:** Rydell classification.

Grade 0	Epidural scar tissue was not adherent to the dura mater
Grade 1	Epidural scar tissue was adherent to the dura mater but easily dissected
Grade 2	Epidural scar tissue was adherent to the dura mater and difficultly dissected without disrupting the dura matter
Grade 3	Epidural scar tissue was firmly adherent to the dura mater and could not be dissected

**Table 2:** Grades of epidural scar adhesion in rats, according to the Rydell standard.

Group^#^	Grade			
	0	1	2	3
*A. sinensis *	3	2	0	0
Saline	0	0	1	4
Sham	0	0	0	5

^#^Five rats were selected from each treatment group. Values within table represent number of rats.

**Table 3:** Grades of cell density.

Groups	Fibroblast density	Inflammatory cell density
*A. sinensis *		
* A. sinensis* 1	1	1
* A. sinensis *2	1	1
* A. sinensis* 3	1	2
* A. sinensis* 4	2	1
* A. sinensis* 5	1	1
Saline		
Saline 1	3	3
Saline 2	3	3
Saline 3	3	3
Saline 4	3	3
Saline 5	2	3
Sham		
Sham 1	3	3
Sham 2	3	3
Sham 3	3	3
Sham 4	3	3
Sham 5	3	3
